# Explaining the low-frequency shear elasticity of confined liquids

**DOI:** 10.1073/pnas.2010787117

**Published:** 2020-08-03

**Authors:** Alessio Zaccone, Kostya Trachenko

**Affiliations:** ^a^Department of Physics “A. Pontremoli,” University of Milan, 20133 Milan, Italy;; ^b^Department of Chemical Engineering and Biotechnology, University of Cambridge, Cambridge CB3 0AS, United Kingdom;; ^c^Cavendish Laboratory, University of Cambridge, Cambridge CB3 0HE, United Kingdom;; ^d^School of Physics and Astronomy, Queen Mary University of London, London E1 4NS, United Kingdom

**Keywords:** liquids, confined liquids, rheology, amorphous materials

## Abstract

Experimental observations of unexpected shear rigidity in confined liquids, on very low frequency scales on the order of 0.01 to 0.1 Hz, call into question our basic understanding of the elasticity of liquids and have posed a challenge to theoretical models of the liquid state ever since. Here we combine the nonaffine theory of lattice dynamics valid for disordered condensed matter systems with the Frenkel theory of the liquid state. The emerging framework shows that applying confinement to a liquid can effectively suppress the low-frequency modes that are responsible for nonaffine soft mechanical response, thus leading to an effective increase of the liquid shear rigidity. The theory successfully predicts the scaling law $G^{\prime }∼L^{-3}$ for the low-frequency shear modulus of liquids as a function of the confinement length L, in agreement with experimental results, and provides the basis for a more general description of the elasticity of liquids across different time and length scales.

The elasticity of liquids is well understood in the high frequency limit of the mechanical response, where pioneering work by Frenkel (1) has shown that the response of a liquid is basically indistinguishable from that of an amorphous solid, provided the frequency of mechanical oscillation is sufficiently high. The idea here is that at short times (high frequency) the diffusive component of the liquid motion is absent and liquids behave as solids. This has become an accepted view (2). However, later experiments have challenged this view (3–7) and found a remarkable solid-like property of liquids to support shear stress at very low frequency, albeit in confinement. This phenomenon is not currently understood. This is a limitation for the full development of small-scale nano-, micro-, and submillimeter flow technologies.

High-frequency mechanical response of liquids is typically measured with ultrasonic techniques in the megahertz range corresponding to shear elastic moduli of the order of gigapascals (8). The behavior is well described by Frenkel’s theory, which links it to transverse acoustic phonons and their vanishing at a characteristic internal time scale, the Frenkel time, which is related to the viscoelastic Maxwell time. Conversely, low-frequency shear elasticity has been identified fairly recently (in view of the long history of liquid research), starting with the pioneering work of Derjaguin et al. (3, 4) and of Noirez and coworkers (6, 7). The low-frequency elasticity of liquids is weaker, on the order of $1-10^{3}$ Pa, and is strongly dependent on the submillimeter confinement length scale of the liquid.

Here we provide a description of liquid elasticity inspired by Frenkel’s ideas on the phonon theory of liquids, combined with recent developments in the microscopic theory of elasticity of amorphous materials. The resulting framework allows us to decompose the various contributions to liquid elasticity based on wavevector k, and thus to identify how the shear modulus of a liquid changes upon varying the confinement length L.

Following previous literature (9), we introduce the Hessian matrix of the system ${\underline{\underline{H}}}_{ij}=-\partial^{2}U/\partial {\underline{\overset{˚}{q}}}_{i}\partial {\underline{\overset{˚}{q}}}_{j}$ and the affine force field ${\underline{\Xi }}_{i,\kappa \chi }=\partial {\underline{f}}_{i}/\partial \eta_{\kappa \chi }$, where $\eta_{\kappa \chi }$ is the strain tensor. Here, ${\overset{˚}{q}}_{i}$ is the coordinate of atom i in the initial undeformed frame (denoted with the ring notation), whereas ${\underline{f}}_{i}=\partial U/\partial {\underline{q}}_{i}$ represents the force acting on atom i in the affine position, that is, in the initial frame subject to macroscopic deformation, hence the name ”affine” force field. Greek indices refer to Cartesian components of the macroscopic deformation (i.e., κχ=xy for shear). For a liquid, the Hessian ${\underline{\underline{H}}}_{ij}$ is typically evaluated in a reference state obtained from averaging over nonfully equilibrated configurations to include instantaneous normal modes (imaginary frequencies) (9).

As shown previously, the equation of motion of atom i, in mass-rescaled coordinates, can be written (9, 10)

$$\frac{d^{2}{\underline{x}}_{i}}{dt^{2}}+\nu \frac{d{\underline{x}}_{i}}{dt}dt+{\underline{\underline{H}}}_{ij}{\underline{x}}_{j}={\underline{\Xi }}_{i,\kappa \chi }\eta_{\kappa \chi },$$ [1]

where $\underline{\underline{\eta }}$ is the Green–Saint Venant strain tensor and ν is a microscopic friction coefficient which arises from long-range dynamical coupling between atoms mediated by anharmonicity of the pair potential. The term on the right-hand side physically represents the effect of the disordered (noncentrosymmetric) environment leading to nonaffine motions: A net force acts on atom i in the affine position (i.e., the position prescribed by the external strain tensor $\eta_{\kappa \chi }$). As a consequence, in order to keep mechanical equilibrium on all atoms throughout the deformation, an additional nonaffine displacement is required in order to relax the force $f_{i}$ acting in the affine position. This displacement brings each atom i to a new (nonaffine) position.

The above equation of motion can be derived from a model particle-bath Hamiltonian as shown in previous work (9). Furthermore, $\left\{{\underline{x}}_{i}\left(t\right)={\underline{\overset{˚}{q}}}_{i}\left(t\right)-{\underline{\overset{˚}{q}}}_{i}\right\}$, as an expansion around a reference state ${\underline{\overset{˚}{q}}}_{i}$. Following standard manipulations, which involve Fourier transformation and eigenmode decomposition from time to eigenfrequency (10), and applying the definition of elastic stress, one obtains the following expression for the complex elastic constants (9, 10):

$$C_{\alpha \beta \kappa \chi }\left(\omega \right)=C_{\alpha \beta \kappa \chi }^{Born}-\frac{1}{V}\underset{n}{\sum }\frac{{\hat{\Xi }}_{n,\alpha \beta }{\hat{\Xi }}_{n,\kappa \chi }}{\omega_{p,n}^{2}-\omega^{2}+i\omega \nu },$$ [2]

where $C_{\alpha \beta \kappa \chi }^{Born}$ denotes the affine part of the elastic constant, that is, what survives in the high-frequency limit. Also, ω denotes the oscillation frequency of the external strain field, whereas $\omega_{p}$ denotes the internal eigenfrequency of the liquid [which results, e.g., from diagonalization of the Hessian matrix (9)]. We use the notation $\omega_{p}$ to differentiate the eigenfrequency from the external oscillation frequency ω.

In liquids, a microscopic expression for $G_{\infty }\equiv C_{xyxy}^{Born}$ is provided by the well-known Zwanzig–Mountain (ZM) formula (11), in terms of the pair potential V(r) and the radial distribution function g(r). The sum over n in Eq. **2** runs over all 3N degrees of freedom (for a monoatomic liquid with central-force pair interaction). Also, we recognize the typical form of a Green’s function, with an imaginary part given by damping and poles $\omega_{p,n}$ which correspond to the eigenfrequencies of the excitations.

As usual when dealing with eigenmodes, the sum over n (labeling the eigenmode number) can be replaced with a sum over wavevector k, with $k^{2}=k_{x}^{2}+k_{y}^{2}+k_{z}^{2}$, and $k_{x}=\pi n_{x}/L$. We then recall that the numerator of the Green’s function, which is given by the eigenfrequency spectrum of the affine force field, can be expressed as $\Gamma \left(\omega_{p}\right)={\left\langle {\hat{\Xi }}_{n,xy}{\hat{\Xi }}_{n,xy}\right\rangle }_{n\in \left\{\omega_{p},\omega_{p}+\delta \omega_{p}\right\}}\approx A\omega_{p}^{2}$, where $A\approx \left(1/15\right)\kappa R_{0}^{2}$ with κ the spring constant for the intermolecular bond and $R_{0}$ the bonding distance, as proved analytically in ref. 12. This parabolic law holds up to high eigenfrequencies as shown in simulations (9).

We thus rewrite Eq. **2** in terms of a sum over k as follows:

$$G^{*}\left(\omega \right)=G_{\infty }-\frac{A}{V}\underset{k}{\sum }\frac{\omega_{p,k}^{2}}{\omega_{p,k}^{2}-\omega^{2}+i\omega \nu },$$ [3]

where A is a numerical prefactor.

In isotropic media, eigenmodes can be divided into longitudinal (L) and transverse (T) modes. Therefore we can split the sum in Eq. **3** into a sum over L modes and a sum over T modes,

$$G^{*}\left(\omega \right)=G_{\infty }-A\underset{k\lambda }{\sum }\frac{\omega_{p,k\lambda }^{2}}{\omega_{p,k\lambda }^{2}-\omega^{2}+i\omega \nu },$$ [4]

where λ=L,T. Furthermore, we introduce continuous variables for the eigenfrequencies $\omega_{p}\left(k\right)$, by invoking appropriate dispersion relations $\omega_{p,L}\left(k\right)$ and $\omega_{p,T}\left(k\right)$ for L and T modes, respectively (as discussed below). Hence, the discrete sum over eigenstates can be replaced, as is standard in solid-state physics, with a continuous integral in k-space, $\sum_{k}\ldots \to \frac{V}{{\left(2\pi \right)}^{3}}\int \ldots d^{3}k$:

$$\begin{array}{ll} G^{*}\left(\omega \right)= & G_{\infty }-B\int_{0}^{k_{D}}\frac{\omega_{p,L}^{2}\left(k\right)}{\omega_{p,L}^{2}\left(k\right)-\omega^{2}+i\omega \nu }k^{2}dk\\ & -B\int_{0}^{k_{D}}\frac{\omega_{p,T}^{2}\left(k\right)}{\omega_{p,T}^{2}\left(k\right)-\omega^{2}+i\omega \nu }k^{2}dk, \end{array}$$ [5]

the upper limit of the integral is set by the Debye cutoff wavevector $k_{D}$, which, in any condensed matter system (be it solid or liquid), sets the highest frequency of atomic vibration.

One should note that while k is in general not a good quantum number in amorphous materials (as the connection between energy and wavevector is no longer single-valued as it is in crystals where Bloch’s theorem holds), it still can be used to provide successful descriptions of the properties of amorphous materials and liquids (13).

We now discuss the dispersion relations for longitudinal and transverse excitations in liquids. For the longitudinal modes, one can resort to the Hubbard–Beeby theory of collective modes in liquids (14), which has been shown to provide a good description of experimental data, and use equation 43 in ref. 14. As will be shown below, the final result for the low-frequency $G^{\prime }$ does not depend on the form of $\omega_{p,L}$. However, for the mathematical completeness of the theory it is important to specify which analytical forms for the dispersion relations can be used.

Differently from the gapless longitudinal dispersion relations and generally from phonon dispersion relations in solids, liquids have the gap in k-space in the transverse phonon sector. This follows from the dispersion relation (15),

$$\omega_{p,T}\left(k\right)=\sqrt{c^{2}k^{2}-\frac{1}{4\tau^{2}}},$$ [6]

where τ is the liquid relaxation time and c is the transverse speed of sound.

Eq. **6** follows from the Maxwell–Frenkel approach to liquids where the starting point of liquid description includes both elastic and viscous response (15) and implies that transverse modes in liquids propagate above the threshold value $k_{g}=\frac{1}{2c\tau }$, thus setting the gap in momentum space, as ascertained on the basis of molecular dynamics simulations in liquids (16). At the atomistic level, the Frenkel theory attributes τ to the average time between molecular rearrangements in the liquid (1). In the limit of large τ or viscosity, Eq. **6** becomes gapless and solid-like.

In a large system, $k_{g}$ sets the infrared cutoff in a sum or integral over k-points. In a confined system with a characteristic size L, the lower integration limit becomes

$$k_{min}=\max\left(k_{g},\frac{1}{L}\right).$$ [7]

Then,

$$\begin{array}{ll} G^{*}\left(\omega \right)= & G_{\infty }-B\int_{\frac{1}{L}}^{k_{D}}\frac{\omega_{p,L}^{2}\left(k\right)}{\omega_{p,L}^{2}\left(k\right)-\omega^{2}+i\omega \nu }k^{2}dk\\ & -B\int_{k_{min}}^{k_{D}}\frac{\omega_{p,T}^{2}\left(k\right)}{\omega_{p,T}^{2}\left(k\right)-\omega^{2}+i\omega \nu }k^{2}dk, \end{array}$$ [8]

where the lower integration limit for the longitudinal modes in the second term is given by the system size L. The lower integration limit for the transverse modes in the third term is given by $k_{min}$ in Eq. **7**. We take the real part of $G^{*}$ which gives the storage modulus $G^{\prime }$ and focus on low external oscillation frequencies $\omega \ll \omega_{p}$ used experimentally. In both integrals numerator and denominator cancel out, leaving the same expression in both integrals. Therefore, as anticipated above, the final low-frequency result does not depend on the form of $\omega_{p,L}\left(k\right)$, nor of $\omega_{p,L}\left(k\right)$, although the latter, due to the k-gap, plays an important role (see Eq. **7**) in controlling the infrared cutoff of the transverse integral. In the experiments where the size effect of confinement is seen, $k_{g}\ll \frac{1}{L}$ (17), and $k_{min}=\frac{1}{L}$ according to Eq. **7**, leading to

$$G^{\prime }=G_{\infty }-\alpha \int_{1/L}^{k_{D}}k^{2}dk=G_{\infty }-\frac{\alpha }{3}k_{D}^{3}+\frac{\beta }{3}L^{-3}.$$ [9]

Here the only term which depends on the system size is the last term, while α and β are numerical prefactors. In a liquid in thermodynamic equilibrium, using the stress-fluctuation version of the nonaffine response formalism [the two versions have been shown to be equivalent (18)] and equilibrium statistical mechanics, it has been shown in ref. 19 that the affine term $G_{\infty }$ and the negative nonaffine term (here, $-\frac{\alpha }{3}k_{D}^{3}$) cancel each other out exactly, such that $G^{\prime }\left(\omega \to 0\right)=0$ for L→∞ (bulk liquids). Therefore, for liquids under submillimiter confinement, only the third term in the above equation survives, and we obtain

$$G^{\prime }\approx \beta^{\prime }L^{-3},$$ [10]

where $\beta^{\prime }=\beta /3$ is a numerical prefactor. It should be noted that $G_{\infty }$ does not depend on L because in, for example, the ZM formula it is given as an integral that contains dV(r)/dr, which is zero after few molecular diameters.

We now compare Eq. **10** to available experimental data of low-frequency $G^{\prime }$ of confined liquids as a function of the confinement length L using the data of the LC short-chain polymer in the isotropic state (note that Eq. **2** has been successfully tested also for polymer melts in ref. 9). In Fig. 1 we compare the trend for the storage modulus $G^{\prime }$ as a function of confinement length L predicted by Eq. **10**, with well-controlled experimental data of confined LC-polymer (${PAOCH}_{3}$) liquids (in the isotropic state), well above the glass transition temperature $T_{g}$, taken from ref. 6. It is evident that the experimental data follow the $L^{-3}$ law predicted in this work. Other experimental systems in the literature are also well compatible with the predicted $G^{\prime }∼L^{-3}$ scaling. These include ionic liquids (20), nonentangled polymer liquids (21), and even nanoconfined water probed by atomic force microscopy such as the data in figure 2(b′) of ref. 5. Also, in the limit L→∞, the above equation Eq. **10** recovers the well-known result for liquids, that is, $G^{\prime }=0$ at low frequency because the third term on the right-hand side vanishes while the first two terms (affine and nonaffine, respectively) cancel each other out exactly in equilibrium liquids as rigorously demonstrated in ref. 19.

**Fig. 1.**
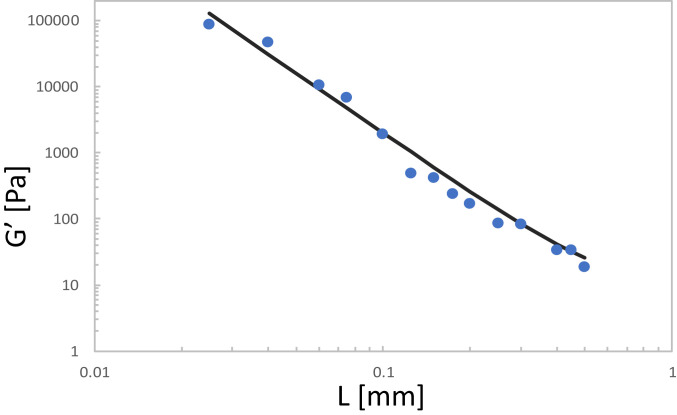
Low-frequency (≈0.01 Hz) storage modulus $G^{\prime }$ as a function of confinement length L. Experimental data refer to short-chain liquid crystalline (LC) polymer liquids ${PAOCH}_{3}$ (in the isotropic state) well above $T_{g}$ (6), whereas the solid line is the prediction from Eq. **10**.

In conclusion, we have developed an analytical theory of the shear modulus of liquids based on nonaffine atomic deformations. This approach allows us to decompose the nonaffine elasticity of the liquid into different phonon-like contributions in terms of their momentum k. Since the overall nonaffine/relaxational contribution to the low-frequency shear modulus is negative, and is expressed as an integral over k, the effect of confinement leads to an infrared (long-wavelength) cutoff of the k-integral. which is inversely proportional to confinement size L. This explains why reducing the confinement size L effectively increases the shear rigidity by suppressing long-wavelength nonaffine relaxations that soften the response. The predicted $G^{\prime }∼L^{-3}$ law is followed by many different experimental systems and may open up new avenues for the controlled manipulation of liquids at the micro and nanoscale (5).

## Data Availability

All study data are included in the article.
